# Modeling Hereditary Angioedema With Personalized Expanded Potential Stem Cell‐Derived Hepatocytes: A CRISPR‐Validated Platform for Mutation‐Specific Mechanisms and Therapeutic Innovation

**DOI:** 10.1111/all.70419

**Published:** 2026-06-20

**Authors:** Xueyan Liu, Yuxin Wang, Jane C. Y. Wong, Xiang Zhang, Cuixi Wu, Pengyu Chen, Yunzhi Yang, Pentao Liu, Chak Sing Lau, Matthew Cook, Philip Hei Li

**Affiliations:** ^1^ Division of Rheumatology and Clinical Immunology, Department of Medicine Queen Mary Hospital, University of Hong Kong Hong Kong SAR China; ^2^ InnoHK Centre for Translational Stem Cell Biology Hong Kong Science Park Hong Kong SAR China; ^3^ School of Biomedical Sciences University of Hong Kong Hong Kong SAR China; ^4^ School of Clinical Medicine University of Cambridge Cambridge UK

## Abstract

Hereditary angioedema (HAE) with C1 esterase inhibitor (C1INH) deficiency is caused by pathogenic *SERPING1* mutations that disrupt production of the plasma protease inhibitor C1INH. However, the molecular mechanisms and consequences of patient‐specific mutations remain poorly understood due to the lack of physiologically relevant human models. Here, we established a personalized, isogenic, stem‐cell‐derived hepatocyte platform to investigate the underlying mutation‐specific mechanisms of HAE. Specifically, peripheral blood mononuclear cell (PBMC)‐expanded erythroblasts from four representative HAE‐C1INH‐Type1 patients containing distinct point, insertion, deletion, or large fragment *SERPING1* mutations were reprogrammed into expanded potential stem cells (EPSCs) and further differentiated into hepatocyte‐like cells (HLCs). These HLCs exhibited appropriate transcriptional transitions, mature hepatic features, and C1INH secretion comparable to that observed in human plasma. All patient‐derived HLCs demonstrated impaired C1INH secretion with mutation‐specific differences in both *SERPING1* transcription and intracellular accumulation. Moreover, to verify that the mutations directly drive the phenotype, we performed CRISPR/Cas9‐mediated genome repair, which restored *SERPING1* mRNA expression and C1INH secretion. Conversely, identical patient mutations installed into healthy EPSCs showed the same transcriptional and secretory defects, confirming sufficiency. Collectively, we have established a robust human hepatocyte model that accurately recapitulates key hepatocyte‐specific aspects of HAE pathophysiology and provides a scalable foundation for investigation of future precision therapies.

## Graphical abstract

Patient‐derived expanded potential stem cell (EPSC) hepatocytes reveal that pathogenic *SERPING1* variants cause distinct cellular defects in hereditary angioedema. While most mutations reduce *SERPING1* transcription and C1‐INH secretion, a large deletion induces intracellular C1‐INH retention. Reciprocal CRISPR repair and installation confirm mutation‐specific causality. CRISPR, clustered regularly interspaced short palindromic repeats; C1‐INH, C1 esterase inhibitor; EPSC, expanded potential stem cell; P, patient; *SERPING1* , serpin family G member 1.

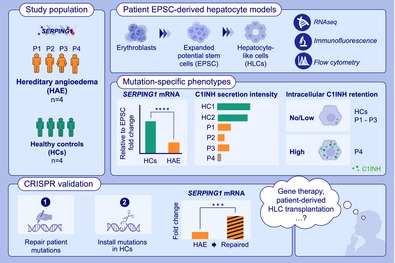

## 1 Introduction

Hereditary angioedema (HAE) is a rare autosomal dominant disorder characterized by recurrent episodes of swelling involving the skin, gastrointestinal tract, and airway [1, 2]. The majority of HAE cases are caused by pathogenic mutations in the *SERPING1* gene, which encodes the protease inhibitor C1 esterase inhibitor (C1INH), a key regulator of the complement, contact, and fibrinolytic pathways [2, 3, 4, 5]. HAE due to C1INH deficiency (HAE‐C1INH‐Type1/Type2) is classified into Type1 (low C1INH levels) and Type2 (normal or elevated but dysfunctional C1INH) [5, 6, 7]. In both types, loss of C1INH activity results in unregulated kallikrein activation and excessive bradykinin production, leading to increased vascular permeability and tissue edema [5]. Although current acute and prophylactic therapies have substantially improved patient outcomes, they do not address the underlying *SERPING1* genetic defect, and the molecular consequences of individual mutations remain incompletely understood [8].

*SERPING1* exhibits extensive genetic heterogeneity. More than 800 pathogenic mutations have been reported to date, including single‐nucleotide substitutions, small insertions or deletions, and large genomic rearrangements [3, 9]. These mutations are believed to contribute significantly to clinical heterogeneity, particularly in disease severity and response to treatment, although no clear genotype–phenotype correlation has been established [10]. Mechanistically, they may reduce transcript abundance, promote misfolded‐protein degradation, impair intracellular trafficking, disrupt serpin folding, or compromise secretion of C1INH. However, defining their exact mechanistic consequences has been limited by the absence of physiologically relevant human models.

Since *SERPING1* is highly expressed in hepatocytes and the liver is the principal source of circulating C1INH, a renewable human hepatocyte model that preserves patient genotypes would be ideal for mechanistic studies of HAE [11]. Prior to the availability of newer therapies, attenuated androgens were commonly used for HAE prophylaxis and remain in use in certain non‐Western and resource‐limited settings. However, prolonged use has been associated with hepatic complications, including non‐alcoholic fatty liver disease and cirrhosis [12, 13, 14, 15, 16]. Notably, some reports suggest that fatty liver may precede androgen use, raising the possibility of intrinsic hepatic involvement in HAE pathophysiology, potentially mediated by *SERPING1* beyond its role in C1INH deficiency [17]. Despite these clinical observations, existing experimental models remain limited.

Previous studies relied on transfected HeLa and HepG2 carcinoma cell lines to demonstrate dominant‐negative effects of specific full‐length *SERPING1* mutations, attributed to intracellular C1INH aggregates [12, 13]. However, hepatoma lines may lack physiological endogenous C1INH expression, and overexpression may artificially exacerbate aggregation phenotypes [12, 14]. Moreover, in these systems, mutant *SERPING1* genes are not situated in their native chromosomal context and lack patient‐specific regulatory elements and epigenetic features. These factors, which differ from those found in normal hepatocytes, can influence protein folding, the endoplasmic reticulum (ER) stress response, and potentially either exaggerate or mask variant effects. Mouse models, meanwhile, are constrained by species‐specific differences in serpin biology, complement regulation, and hepatocyte gene expression, further underscoring the need for a human‐relevant, patient‐derived system [18, 19].

Recent advances in stem cell technologies now enable the generation of patient‐specific hepatocyte platforms for HAE. Patient‐derived stem cells retain the full genetic background, offering a more physiologically faithful model for studying disease mechanisms and therapeutic responses [20]. To date, there are no stem cell models in HAE. Expanded potential stem cells (EPSCs) offer several key advantages for studying diseases like HAE, where subtle changes in transcription or protein folding critically affect C1INH levels [21]. First, EPSCs can be efficiently derived from patient peripheral blood mononuclear cell (PBMC)‐expanded erythroblasts and differentiated into all cell lineages while maintaining stable pluripotency and genetic stability [22]. Second, because patient‐derived EPSCs preserve the full genetic background, they can capture mutation‐specific effects and allow direct evaluation of endogenous *SERPING1* regulation, which are processes that cannot be reliably modeled in non‐patient or cancer‐derived cell lines. In addition, patient‐derived EPSCs are fully compatible with precise genome editing, allowing direct testing of variant pathogenicity through both correction and introduction of mutations.

In this study, we established a patient‐specific human EPSC‐hepatocyte platform to investigate the molecular consequences of *SERPING1* mutations in HAE. Using peripheral blood from four patients harboring distinct pathogenic mutations (including point, insertion, deletion, and large‐fragment genomic deletions) we generated patient‐specific EPSC lines and differentiated them into HLCs. We then characterized their transcriptional profiles, protein quantification, intracellular C1INH distribution, and secretory function. To establish causality, we used CRISPR/Cas9 to repair patient mutations and assessed whether C1INH production was rescued. Conversely, we introduced the same mutations into healthy EPSCs to determine whether these single genetic alterations were sufficient to recapitulate the disease phenotype.

## 2 Methods

### 2.1 Patient Selection

All HAE patients were diagnosed in accordance with international guidelines [2]. PBMCs were isolated from the peripheral blood of HAE patients and healthy controls (HCs) for subsequent processing. All blood donors provided informed consent, and the study protocol was approved by the Institutional Review Board of the University of Hong Kong/Hospital Authority Hong Kong West Cluster. The mutation site of each patient's *SERPING1* protein was visualized using PyMOL 3.1.

### 2.2 EPSC Reprogramming, Maintenance and Characterization

EPSC reprogramming, maintenance, and characterization were performed using PBMC‐expanded erythroblasts from patient or HCs, as described in our previous study, with the exception that the erythroblast expansion medium was replaced by StemSpan SFEM II (STEMCELL Technologies) basal medium supplemented with 300 ng/mL SCF (Peprotech), 50 ng/mL IL‐3 (Peprotech), and 100 ng/mL EPO (Peprotech) [22]. To confirm the genotype of each EPSC line, genomic DNA containing the *SERPING1* mutations was amplified via PCR and subsequently analyzed by Sanger sequencing using the amplify or sequence primers listed in Table S1. The pluripotency of EPSCs was assessed by quantitative real‐time PCR (qRT‐PCR), flow cytometry, and immunofluorescence staining for the pluripotency markers *OCT4*, *SOX2*, and *NANOG*. Trilineage differentiation potential of EPSCs was evaluated by embryonic body formation and immunofluorescence staining for the trilineage markers *SOX17* (endoderm), SMA (mesoderm), and β‐tubulin (ectoderm). Primer sequences for qRT‐PCR and antibodies used for flow cytometry and immunofluorescence staining are listed in Tables S1 and S2. Experimental procedures were conducted following techniques described in previous studies [22].

### 2.3 EPSC‐Derived HLC Differentiation and Characterization

Differentiation of HLCs from patient or control EPSCs was conducted using the STEMdiff Hepatocyte Kit (STEMCELL Technologies), with minor modifications to the manufacturer's protocol. Briefly, EPSCs were seeded at approximately 80% confluency on a Laminin‐521 (StemCell Technologies) coated 8‐well chamber slide (Ibidi) or 12‐well plate and cultured overnight in EPSC medium supplemented with 10 μM Y‐27632 (Tocris). The EPSC medium was sequentially switched to definitive endoderm medium on Day 1, hepatic progenitor medium on Day 5, and hepatocyte medium on Day 10. The medium was changed daily before Day 10 and every 2 days from Day 10 to 20, with 500 μL (for 12‐well plate) or 300 μL (for 8‐well chamber slide) used throughout the study to maintain consistency. Morphological changes at each differentiation stage were documented using photographs. The expression of stage‐specific marker genes was assessed via qRT‐PCR throughout HLC differentiation. These markers included the pluripotency marker *OCT4*; definitive endoderm markers *FOXA2* and *CXCR4*; hepatic progenitor markers *AFP*, *HNF1B*, *HNF6*, *TBX3*, *CK19*, and *HNF4A*; and hepatocyte markers *ALB*, *CYP3A4*, *A1AT*, and *ASGR1*. As a reference, the expression of these markers in the human hepatoblastoma cell line HepG2 was also evaluated. At the end stage of HLC differentiation, immunofluorescence staining was performed to confirm hepatocyte identity, using Epithelial Cell Adhesion Molecule (EpCAM), albumin (ALB), and Alpha‐1 antitrypsin (A1AT) as markers. Concurrently, HLC culture supernatants were collected and analyzed using functional assays for ALB secretion and urea synthesis.

Bulk RNA sequencing was employed to compare the transcriptional profiles of EPSCs, EPSC‐derived HLCs, and primary human hepatocytes (PHHs). PHH RNA‐seq datasets were downloaded from the Gene Expression Omnibus (GEO accession numbers: GSM7908779, GSM7908780, GSM7908778, GSM7908781). To identify differentially expressed genes (DEGs) between EPSCs and EPSC‐derived HLCs, GSEA was performed using the Kyoto Encyclopedia of Genes and Genomes (KEGG) pathway database. Hepatocyte‐specific functional and pathway genes were subsequently extracted and comparatively analyzed across the three cell types (EPSCs, EPSC‐derived HLCs, and PHHs) to assess differentiation efficiency and functional maturation.

### 2.4 Evaluation of *SERPING1* Gene, Protein Expression, Secretion and Cellular Localization

*SERPING1* expression was comprehensively evaluated at both the transcriptional and translational levels across HepG2 cells, PHHs, and EPSC‐derived HLCs. Transcriptional dynamics were assessed longitudinally via qRT‐PCR analysis on Day 0, 5, 10, and 20 of HLC differentiation. To evaluate protein‐level expression, immunoblotting was performed throughout the study. Functional secretion was quantified by measuring C1INH protein levels in HLC culture supernatants and cell pellets, which were then compared to serially diluted human plasma using ALB as a reference marker. Intracellular localization of C1INH was determined using immunofluorescence co‐staining with the hepatic membrane marker EpCAM to precisely visualize spatial distribution. Finally, flow cytometry was utilized to quantify the proportions of ALB‐ and A1AT‐expressing cells, enabling comparative phenotypic assessment between HC and patient‐derived HLCs.

We further compared (1) patient‐derived versus isogenic‐corrected (“repaired”) HLCs and (2) healthy versus engineered patient mutation HLCs, examining intracellular C1INH on Day 18/20 and secreted protein at 48‐h intervals (Days 12–20). All qRT‐PCR primers and immunoblotting antibodies are detailed in Tables S1 and S2, with β‐actin as the housekeeping control. Immunoblotting was performed based on our established protocol [22] except that supernatants were clarified by centrifugation (300 × *g*, 5 min) prior to analysis.

### 2.5 CRISPR/Cas9‐Mediated Isogenic Engineering of *SERPING1*

CRISPR/Cas9 genome editing was employed to: (1) generate isogenic mutation‐corrected EPSC lines from patient‐derived EPSCs, and (2) introduce patient‐specific *SERPING1* isogenic mutations in healthy EPSCs, following the methodology described in our previous *STAT1* gain‐of‐function EPSC study [22]. The single‐stranded guide RNAs (sgRNAs), single‐stranded oligodeoxynucleotides (ssODNs), and genotyping primers used for these genetic modifications are detailed in Table S1.

### 2.6 Allele Specific Expression (ASE) and Splicing Analysis of *SERPING1*

To compare allele‐specific mutant expression of *SERPING1*, ASE analysis was performed using bulk RNA sequencing data from HC‐derived, patient‐derived, and P1 isogenic EPSC‐derived HLCs. To visualize mutation‐associated splicing events, BAM alignment files were examined using Integrative Genomics Viewer (IGV).

### 2.7 Statistical Analyses

Statistical analyses were performed using GraphPad Prism (version 9.3.1). *SERPING1* mRNA expression comparisons were assessed using the Mann–Whitney *U* test. Data are presented as median ± interquartile range (IQR). Statistical significance thresholds are denoted as: *p < 0.05, **p < 0.01, ***p < 0.001.

## 3 Results

### 3.1 Patient Selection

**FIGURE 1:**
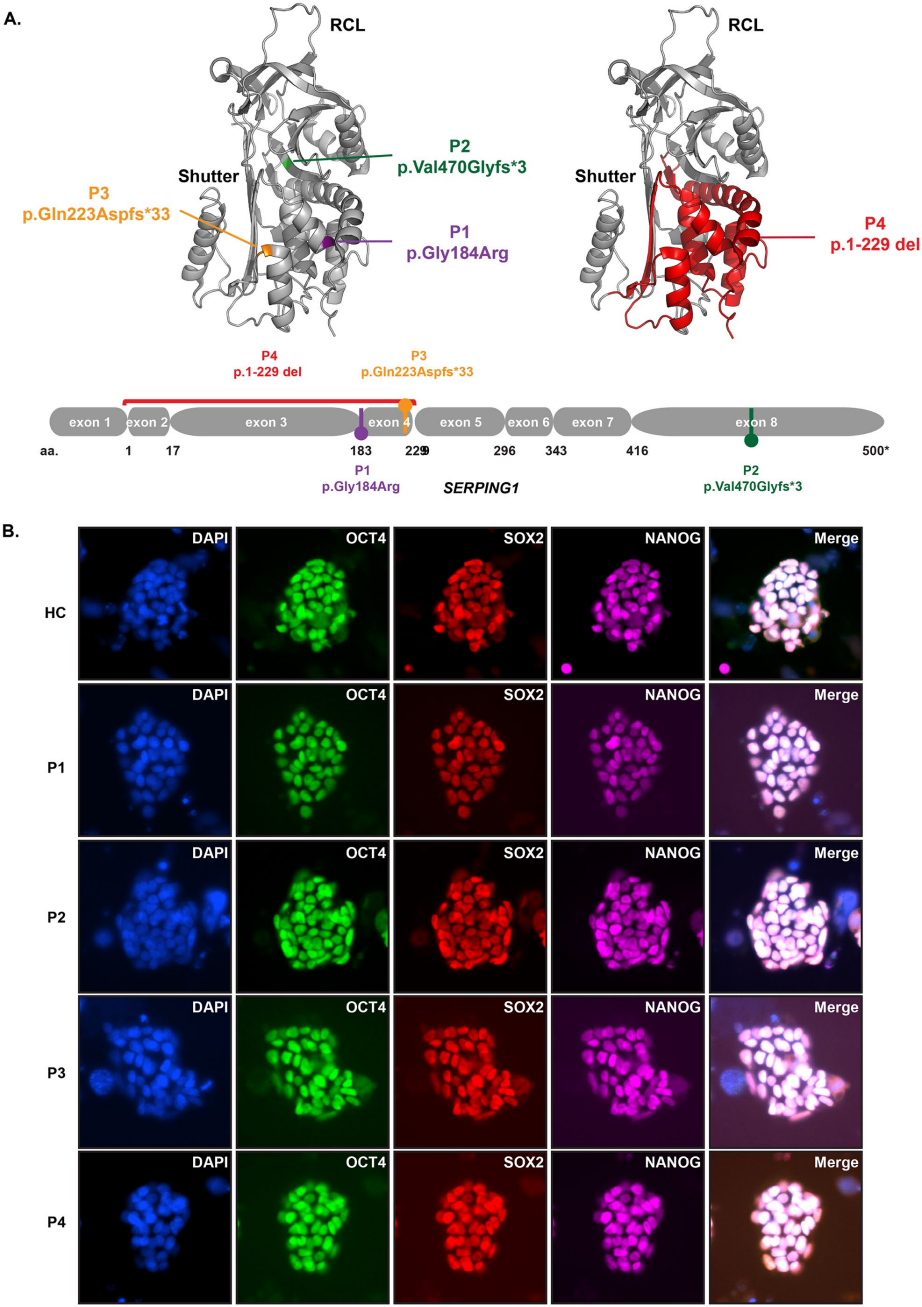
Generation of EPSCs from HAE‐C1INH‐Type1 patients. (A) Illustration of patient‐specific mutations in *SERPING1*. (B) Immunofluorescence staining showing expression of pluripotency markers *OCT4*, *SOX2*, and *NANOG* in EPSCs. HC, healthy control.

PBMCs were obtained from eight individuals: four representative patients with confirmed HAE‐C1INH‐Type1 and four HCs. The HAE patients included P1 (a single‐base pair substitution), P2 (a 1 bp duplication), P3 (a 2 bp deletion), and P4 (a large deletion in *SERPING1* gDNA). Clinical characteristics are summarized in Table 1, genetic, molecular and mutation classification data in Table 2, and mutation locations within exons in Figure 1A.

**TABLE 1:** Clinical characteristics of selected HAE‐C1INH‐Type1 patients and healthy controls.

Patient	HAE type	Age/sex	Clinical characteristics					Laboratory test		
			Age of onset	Location of angioedema	Trigger	History of ICU admission	Prophylactic treatment	C4 (9‐35mg/dL)	C1INH level (21.4–38.7 mg/dL)	C1INH function (0.7–1.3 U/mL)
P1	I	51/Male	21	Limb, lip, larynx	Trauma Dental procedure	Yes	Garadacimab	< 8	8.1 (L)	0.26 (L)
P2	I	47/Male	20	Limb, abdominal, larynx	Trauma	Yes	Tranexamic acid, danazol	< 8	6 (L)	0.33 (L)
P3	I	35/Female	16	Limb, abdominal, larynx	Dental extraction, pressure	No	Garadacimab	9	5.7 (L)	0.2 (L)
P4	I	48/Male	28	Limb, larynx	None	Yes	None	< 8	4.7 (L)	0.18 (L)
HC1	N/A	32/Female	N/A	N/A	N/A	No	N/A	31	28.2	1.57
HC2	N/A	40/Female	N/A	N/A	N/A	No	N/A	29	23.3	1.16
HC3	N/A	25/Female	N/A	N/A	N/A	No	N/A	25	23.6	1.16
HC4	N/A	31/Male	N/A	N/A	N/A	No	N/A	31	29.8	1.49

*Note:* Reference ranges are shown in the column headers.

Abbreviations: L, low; N/A, not applicable.

**TABLE 2:** Genetic, molecular, and mutation classification features of selected HAE‐C1INH‐Type1 patients.

Patient	^1^Transcript change	^2^Nucleotide change	^3^Protein change	Exon localization	Molecular consequence	Reported?	^4^Mutation classification	Functional studies?
P1	c.550G>A	g.7824G>A	p.Gly184Arg	Exon 3	Missense mutation at splice site	Yes [23, 24, 25, 26, 27, 28]	Pathogenic	Yes [12, 28]
P2	c.1408dup	g.21933dup	p.Val470Glyfs*3	Exon 8	Frame shift mutation	No, although other frameshift/nonsense mutations at codon 470 or downstream have been classified as pathogenic/likely pathogenic in ClinVar (p.Val470fs, p.Arg494Ter)	Likely pathogenic	No
P3	c.666‐667delTC	g.9597_9598delTC	p.Gln223Aspfs*33	Exon 4	Frame shift mutation	Yes [24]	Pathogenic	No
P4	—	g.1570_12652del	—	Exon 1–4	Gross deletion	No, but a study reported an exon 1–4 deletion without reporting the exact breakpoint [29]	Pathogenic	No

*Note:* Reference sequence used: ^1^NM_00062.2, ^2^NG_009625.1, ^3^NP_000053.2, ^4^Mutation classification in accordance with the American College of Medical Genetics (ACMG) guidelines [30].

### 3.2 Generation of EPSCs From Patients With HAE

We previously established a protocol to reprogramme patient PBMC‐expanded erythroblasts into EPSCs [22]. Here, the same method was used to establish HAE EPSC, except that an in‐house mixture of SCF, IL‐3, and EPO was used for erythroblast expansion (Figure S1A–C). The success rate of EPSC colony formation ranged from 6 to 17 for each patient. Three colonies of each patient were expanded for further characterization (Figure S1D). All EPSCs expressed endogenous markers of pluripotency, as demonstrated by immunofluorescence staining of *OCT4*, *SOX2* and *NANOG* (Figure 1B). The patient‐specific mutations were subsequently confirmed in each corresponding EPSC line. Immunofluorescence analysis further confirmed successful differentiation into all three germ layers as evidenced by strong nuclear expression of the endodermal marker *SOX17*, prominent filamentous staining for mesodermal marker SMA and a network of mature, post‐mitotic neurons stained positive for β‐III tubulin confirming ectodermal differentiation (Figure S1E).

### 3.3 Generation of HLCs From EPSCs Derived From HAE Patients and HCs

**FIGURE 2:**
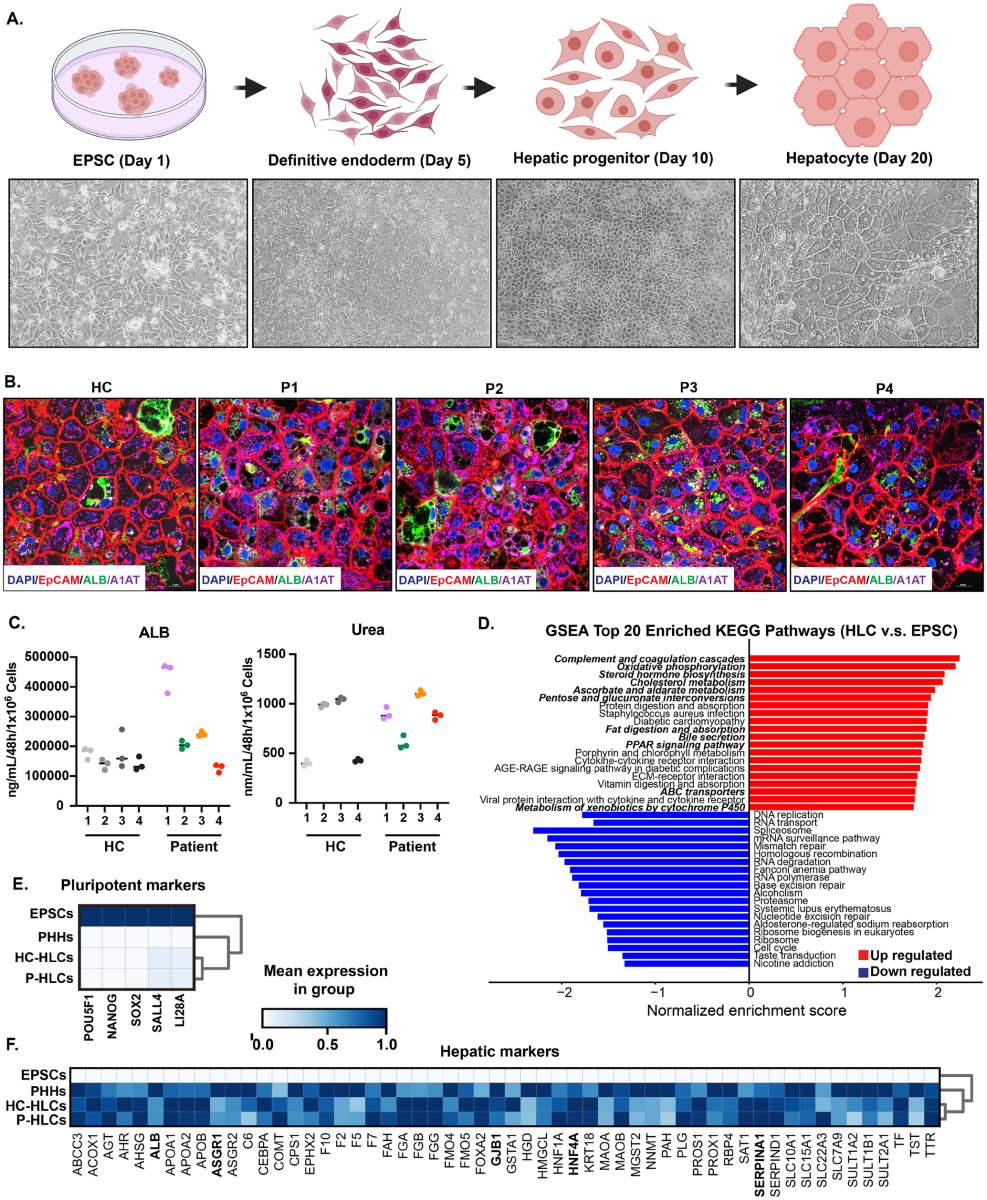
Characterization of HLCs differentiated from EPSCs. (A) Schematic diagram of HLC differentiation, with representative images illustrating each stage. (B) Immunofluorescence staining of hepatocyte marker EpCAM, ALB and A1AT in EPSC‐derived HLCs (200× magnification). (C) Quantification of secreted ALB and synthesized urea in culture supernatants collected at Day 20 from patient‐ or HC‐derived HLCs in 12‐well plates. (D) A list of the top 20 most significantly enriched KEGG pathways identified from DEGs between EPSCs and EPSC‐derived HLCs by bulk RNA sequencing. Red and blue bars indicate pathways significantly up‐regulated and down‐regulated, respectively, in EPSC‐derived HLCs compared to EPSC. Hepatocyte‐specific functional and signaling pathways are highlighted in bold italics for emphasis. (E) and (F) Expression patterns of representative pluripotency and hepatic markers in EPSCs, EPSC‐derived HLCs, and PHHs. HC, healthy control; HC‐HLCs, healthy control‐derived HLCs; PHHs, primary human hepatocytes; P‐HLCs, patient‐derived HLCs.

As previously discussed, the liver plays a crucial role in the pathogenesis of HAE as it is the primary site of C1INH synthesis. Our main objective was therefore to develop an HAE‐specific liver model that would allow us to dissect the contribution of *SERPING1* mutations within a controlled, isogenic context. EPSCs were differentiated into HLCs using an adapted three‐step protocol, sequentially generating definitive endoderm, hepatic progenitors, and mature hepatocytes. Across both HC and patient‐derived EPSC lines, efficient differentiation into HLCs was achieved, with stage‐appropriate morphology observed at each step. A differentiation timeline and representative images from each stage are presented in Figure 2A. The classical polygonal structures and multinucleation of HLCs were evident by phase contrast imaging at the mature stage of HLCs (Figure 2A).

### 3.4 Characterization and Transcriptomic Profile of HLCs From EPSCs

Differentiation efficiency of HLCs was further verified in both HCs and patient cell lines by qRT‐PCR analysis of different stage‐specific markers on Day 0, 5, 10 and 20, and compared to the human hepatoblastoma cell line HepG2 (Figure S2). On Day 0, pluripotent EPSCs showed high expression of *OCT4*, with minimal expression of lineage‐specific markers. On Day 5, co‐expression of *FOXA2* and *CXCR4* was observed. *FOXA2*, a key regulator of endoderm specification, continued to rise through Day 10, whereas *CXCR4*, a key chemokine for endodermal cells, peaked on Day 5 and rapidly downregulated to baseline on Day 10. Differentiation toward hepatic progenitors began around Day 5 and reached its highest levels on Day 10. As cells matured into HLCs, expression of *ALB*, *CYP3A4*, *A1AT*, *ASGR1* continued to increase through Day 20, while hepatic progenitor markers decreased over the same period. An exception was *AFP*, which remained elevated in most cell lines. Gene expression of all transcription factors, with exception of *OCT4* and *CXCR4* were highly expressed in the HepG2 line. Notably, *HNF6*, a transcription factor promoting cholangiocyte lineage was elevated in the HepG2 lines, whereas its expression decreased in EPSC‐derived HLCs by Day 20. Immunofluorescence staining of Day 20 HLCs further confirmed expression of the hepatocyte functional markers ALB and A1AT, along with EpCAM. Strong cytoplasmic ALB and A1AT signals were observed in both control‐ and patient‐derived mature HLCs (Figure 2B). Moreover, both patient and control EPSC‐derived HLCs demonstrated functional ALB secretion and urea synthesis in their culture supernatants (Figure 2C).

RNA‐seq profiling confirmed the hepatic identity of EPSC‐derived HLCs. Gene set enrichment analysis (GSEA) revealed upregulation of liver metabolic and biosynthetic pathways, accompanied by downregulation of proliferation‐ and pluripotency‐associated programs (Figure 2D). Consistently, the global transcriptional profiles of EPSC‐derived HLCs resembled those from PHHs as demonstrated by the upregulation of representative hepatic related genes, and the absence of pluripotent genes (Figure 2E,F; Figure S3A–E). However, gene expression profiles still differed between PHHs and EPSC‐derived HLCs, and the consistent expression patterns observed between HC and patient‐derived HLCs underscore the value of using individual‐specific EPSCs rather than generic cell lines for modeling HAE. Collectively, transcriptional changes, immunofluorescence staining, RNA‐sequencing results, and functional assays demonstrate that EPSCs derived from both HCs and patients can be successfully differentiated into functional HLCs.

### 3.5 Confirmation of *SERPING1* Expression in HLCs and Demonstration of Impaired *SERPING1* Transcript Expression in Patient‐Derived HLCs

**FIGURE 3:**
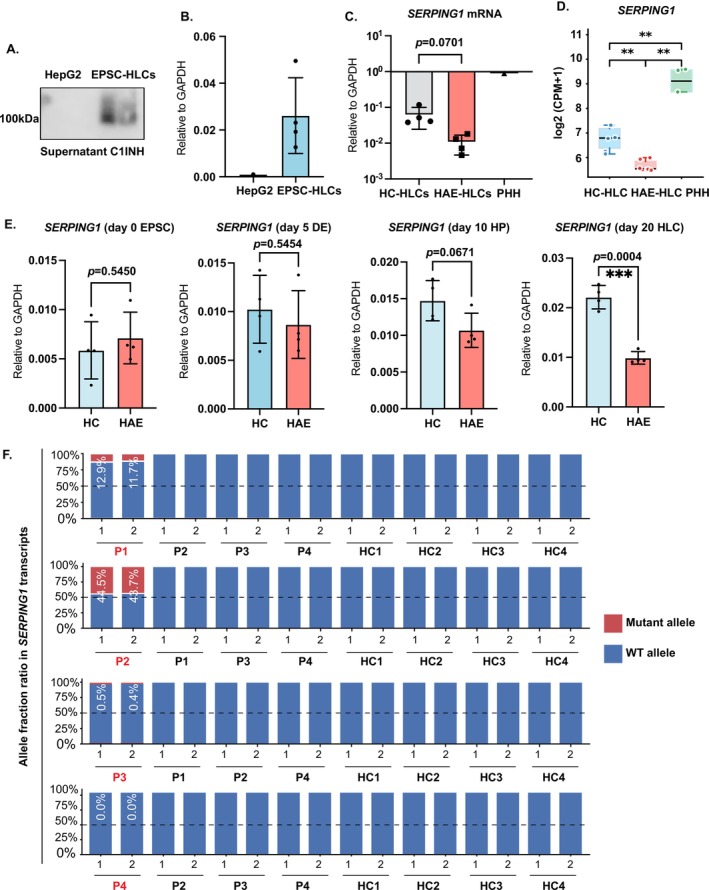
*SERPING1* expression established in healthy EPSC‐derived HLCs and impaired *SERPING1* gene expression in patient HLCs. (A) C1INH protein levels in culture supernatants from Day 20 HC‐derived HLCs and HepG2 cells. (B) RT‐qPCR analysis of *SERPING1* in HC‐derived HLCs and HepG2 cells (*n* = 4). (C‐D) *SERPING1* expression in EPSC‐derived HLCs and PHHs measured by (C) RT‐qPCR (*n* = 4) and (D) bulk RNA sequencing (*n* = 3). (E) Stage‐specific analysis of *SERPING1* expression during HLC differentiation from patient and HC EPSCs. Data are presented as median ± interquartile range (IQR). Statistical significance was determined using the Mann–Whitney *U* test. (F) ASE analysis on *SERPING1* of Day 20 patient and HC HLCs derived from bulk RNA sequencing data.

Given that untransfected HepG2 cells produce negligible levels of C1INH, they were used as a negative control. This allowed us to confirm both the upregulation of *SERPING1* mRNA and the presence of secreted C1INH protein (~100 kDa) in the supernatant of mature EPSC‐derived HLCs, which were not detected in HepG2 cells (Figure 3A,B).

Further comparison with PHHs showed that, although EPSC‐derived HLCs expressed lower levels of *SERPING1* mRNA, patient‐derived HLCs showed a trend toward reduced *SERPING1* expression (Figure 3C). A similar pattern was observed when transcriptomic data from HC and HAE EPSC‐derived HLCs were compared with published PHH transcriptional profiles (Figure 3D). Further stage‐specific analysis revealed defective upregulation of *SERPING1* mRNA during differentiation (Figure S4A), resulting in a markedly reduced fold increase in *SERPING1* expression in patient‐derived HLCs relative to EPSCs compared with controls (Figure S4B). Notably, the most pronounced reduction in *SERPING1* expression was observed at the mature HLC stage (Figure 3E), indicating a differentiation‐stage–specific defect rather than a general transcriptional deficiency across cell types. To further investigate allele‐specific expression, an ASE assay was performed. This analysis demonstrated that the mutant allele was expressed at substantially lower levels than the wild‐type allele across all patient samples, particularly in P1, P3, and P4 (Figure 3F).

### 3.6 Impaired C1INH Secretion and Retention in Patient‐Derived HLCs

**FIGURE 4:**
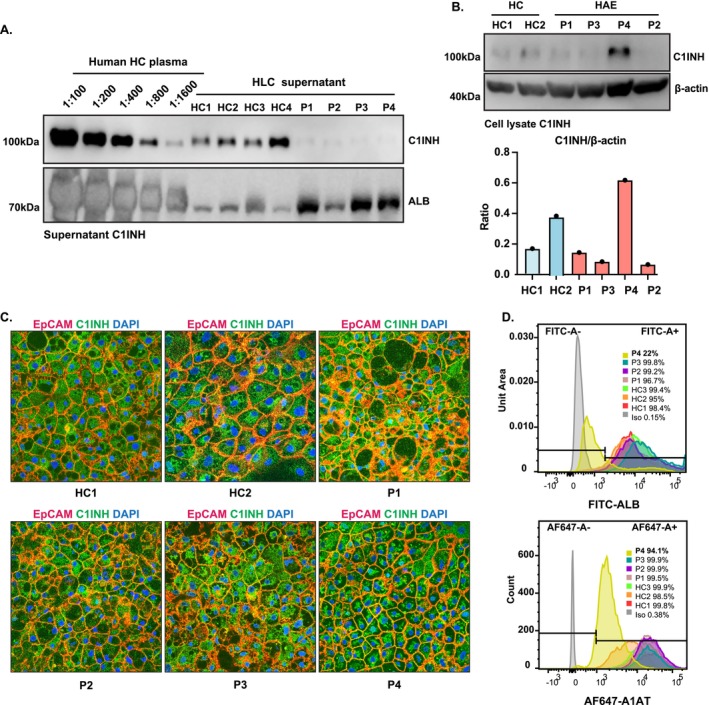
Impaired C1INH secretion and intracellular accumulation in patient HLCs. (A) Analysis of secreted C1INH in serially diluted human plasma and HLC culture supernatants by Western blot. ALB was used as a reference control. (B) Intracellular C1INH protein levels in patient and HC HLC cell pellets, with densitometric quantification relative to β‐Actin shown below. (C) Immunofluorescence staining of C1INH and the hepatic marker EpCAM in control and patient HLCs. (D) Flow cytometric quantification of ALB+ and A1AT+ cell populations in HC and patient HLCs. EPSC‐HLCs, EPSC‐derived HLCs; HC, healthy control.

To investigate C1INH secretion by EPSC‐derived HLCs and to compare its relative abundance with that in human plasma, HC, and patient‐derived HLCs, we performed Western blot analysis to assess both protein size and abundance. Both C1INH and ALB were readily detectable in human plasma and in the supernatants of HLC cultures (Figure 4A). All EPSC‐derived HLCs secreted C1INH at an apparent molecular weight of ~105 kDa, consistent with the heavily glycosylated, active form observed in human plasma. Other reported forms of C1INH, including cleaved (~95–96 kDa), unglycosylated (~55 kDa), and high‐molecular‐weight complexed forms (~160 to > 200 kDa), were not detected (Figure 4A).

Quantitative comparison indicated that the amount of C1INH secreted by control HLCs corresponded to approximately a 1:400–1:800 dilution of human plasma. In contrast, patient‐derived HLCs secreted markedly lower levels of C1INH, corresponding to less than a 1:1600 dilution of plasma, despite retaining robust ALB secretion (Figure 4A; Figure S4C). Intracellular C1INH levels remained low in both HCs and patient lines P1–P3 (Figure 4B). In contrast, P4 exhibited pronounced intracellular accumulation of C1INH accompanied by reduced secretion, suggesting a defect in protein folding or intracellular trafficking (Figure 4B). To further assess intracellular localization, immunofluorescence staining was performed. HLCs derived from HCs and patients P1–P3 displayed a relatively uniform cytoplasmic distribution of C1INH, whereas P4‐derived HLCs exhibited a distinct pattern of intracellular accumulation (Figure 4C). Consistent with this observation, flow cytometry analysis revealed a reduced proportion of ALB‐ and A1AT‐positive cells in P4, indicating a potential defect in HLC maturation, which may also contribute to the observed impairment in C1INH secretion in P4 (Figure 4D).

### 3.7 Restoration of *SERPING1* Expression and C1INH Secretion in CRISPR/Cas9 Repaired HLCs

**FIGURE 5:**
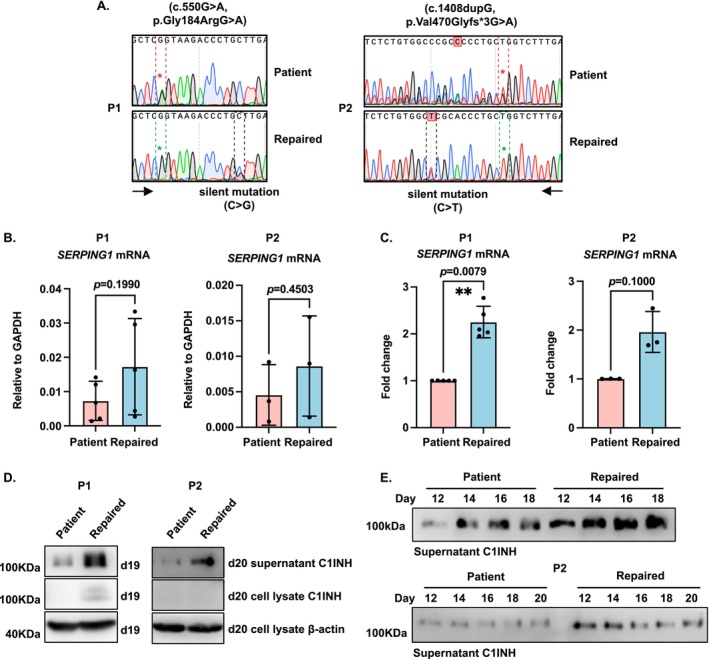
Genetic correction restores *SERPING1* expression and secretion in patient‐derived HLCs. (A) Genotyping analysis of isogenic mutation‐corrected patient EPSCs (labeled as “repaired”). Red asterisks denote patient‐specific mutations; green asterisks indicate corrected base pairs. The black box highlights a silent CRISPR/Cas9‐introduced mutation (non‐amino acid altering) to prevent re‐editing. The black arrow indicates the sequencing direction. (B) Quantitative *SERPING1* mRNA levels and (C) fold change in expression in patient‐derived versus corrected HLCs at differentiation Day 20 (*n* ≥ 3 differentiation batches). Data are presented as median ± interquartile range (IQR). Statistical significance was determined using Mann–Whitney *U* test. (D) Comparative analysis of C1INH protein distribution in culture supernatants and cell lysates between patient‐derived and corrected HLCs at differentiation Day 19 or 20. (E) Time‐course analysis of secreted C1INH (measured by Western blot) in culture supernatants during HLC differentiation.

To demonstrate causality while eliminating confounding inter‐individual genetic variation, we repaired the pathogenic mutations in patient‐derived cell lines using CRISPR/Cas9 and compared the corrected clones to the original, unedited isogenic lines. Patient cell lines P1 and P2 were selected for subsequent analysis as they each harbored a distinct point mutation in the *SERPING1* gene (Figure 5A). Following correction, we observed a significant increase and restoration of *SERPING1* mRNA expression in the repaired HLCs compared to the original unedited lines in both P1 and P2 (Figure 5B,C) through multiple batches of independent differentiation experiments. Functionally, C1INH secretion increased markedly in repaired clones, confirming recovery of protein secretion (Figure 5D). Intracellular C1INH remained minimally detectable in both the original and repaired lines for P1 and P2, consistent with efficient secretion rather than intracellular retention. The increase in extracellular C1INH was further corroborated by Western blot during HLC maturation, with detectable protein as early as Day 12 (Figure 5E).

### 3.8 Engineered *SERPING1* Mutations in Healthy HLCs Recapitulate Deficient C1INH Expression and Secretion

**FIGURE 6:**
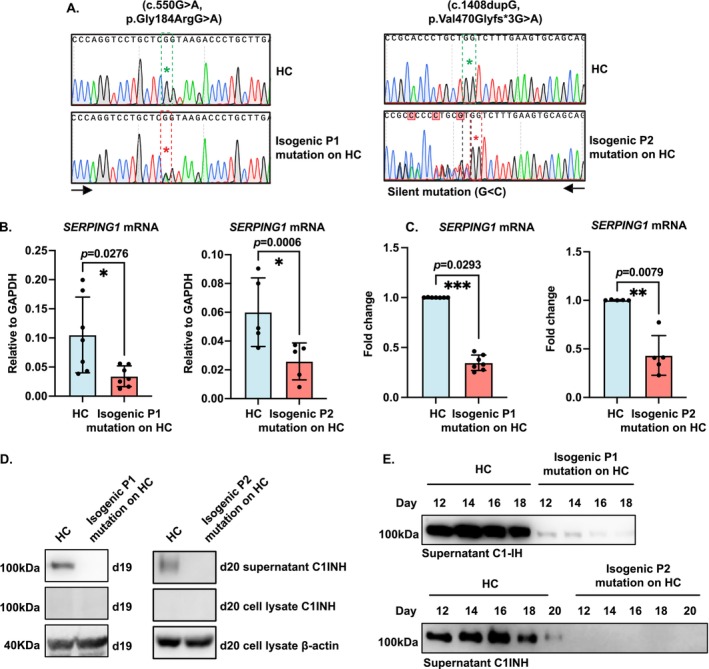
Defective *SERPING1* expression and secretion in isogenic mutation‐installed HC‐derived HLCs. (A) Isogenic installation of patient mutations in healthy EPSCs was validated by Sanger sequencing. Patient‐specific mutations are indicated by red asterisks, while the corresponding wild‐type loci in healthy *SERPING1* are marked with green asterisks. A silent mutation (black box) was introduced to prevent re‐editing. The black arrow indicates the sequencing direction. (B) *SERPING1* gene expression and (C) the fold change analysis in healthy and isogenic mutation‐installed HC‐HLCs on Day 20 of differentiation (*n* ≥ 5 differentiation batches). Data are presented as median ± interquartile range (IQR). Statistical significance was determined using the Mann–Whitney *U* test. (D) C1INH detection in both culture supernatants and cell pellet fractions of healthy and isogenic mutation‐installed HC‐HLCs at differentiation Day 18 or 20 by Western blot. (E) Time‐course analysis of C1INH levels in culture supernatants during HLC differentiation via Western blot. HC‐HLCs, healthy control‐derived HLCs.

To determine whether *SERPING1* mutations were sufficient to drive the disease phenotype, we performed a reciprocal experiment by introducing the same patient‐specific pathogenic mutations into HLCs from HCs, thereby generating isogenic mutant HLCs. Using CRISPR/Cas9, the P1 and P2 mutations were introduced into HC EPSCs, validated by Sanger sequencing, and then differentiated into HLCs (Figure 6A). Consistent with our earlier findings in patient‐derived HLCs, *SERPING1* mRNA levels were significantly reduced in isogenic mutant HLCs compared to their HC counterparts, with an approximately 50% decrease (Figure 6B,C). Functionally, the mutant lines recapitulated the C1INH secretory defect observed in patient‐derived HLCs, with markedly lower extracellular C1INH protein (Figure 6D). This impairment in secretion was already evident by Day 12 of differentiation (Figure 6E).

### 3.9 Genetic Background Modulates *SERPING1* Expression

**FIGURE 7:**
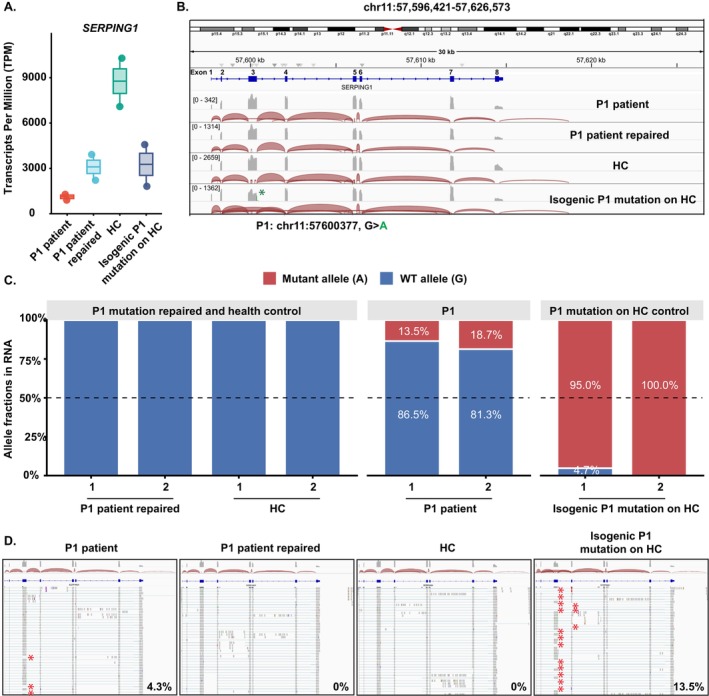
Genetic background alters *SERPING1* expression in P1 isogenic HLCs. (A) Normalized *SERPING1* transcript counts in P1 isogenic HLCs, as determined by bulk RNA sequencing. (B) Visualization of BAM alignment files at the *SERPING1* genomic locus using IGV. The green star indicates the mutant “A” allele located at the terminal base pair of exon 3 (chr11:57600377). (C) ASE analysis of *SERPING1* utilizing bulk RNA sequencing data from the isogenic HLC lines (*n* = 2 differentiation batches). (D) Representative schematic overview of exon skipping events in the original P1 HLCs compared to the mutation‐corrected HC HLCs. Exon skipping sites are indicated by red stars, accompanied by the calculated ratios of exon 3 skipping.

To investigate how individual genetic background influences the functional consequences of a specific mutation, we performed bulk RNA sequencing on HLCs derived from P1 isogenic EPSCs. Transcriptional analysis consistently demonstrated that correction of the P1 mutation restored *SERPING1* expression in patient‐derived HLCs, whereas introduction of the same mutation into the HC background reduced *SERPING1* expression (Figure 7A). Notably, *SERPING1* expression in the corrected P1 HLCs remained lower than the baseline levels observed in the HC cell line (Figure 7A), suggesting an additional influence of genetic background on gene expression. Visualization of BAM alignment files using IGV revealed distinct *SERPING1* expression patterns depending on genetic context. In the HC cell line engineered with the P1 mutation, the mutant ‘A’ allele was predominantly expressed and was associated with prominent exon skipping, particularly at exon 3 (Figure 7B). ASE analysis further highlighted this contrast. In the original P1 line, the mutant allele was expressed at relatively low levels (13.5% and 18.7%), whereas in the mutation‐engineered HC cell line, the mutant allele accounted for nearly all transcripts (95.2% and 100% across replicates) (Figure 7C). This increased mutant allele expression was associated with a higher frequency of exon skipping, predominantly involving exon 3 and occasionally exon 4, reaching up to 13.5% in the engineered HC line compared with 4.3% in P1‐derived HLCs (Figure 7D).

## 4 Discussion

Our study addresses a central challenge in HAE research: mechanistic investigation of the disease has long been limited by the lack of physiologically relevant human models that preserve patient‐specific genomic contexts [18, 31]. To overcome this limitation, we established a patient‐derived, isogenic EPSC‐derived hepatocyte platform that enables mechanistic interrogation of *SERPING1* pathogenic variants in a native human cellular context. By combining patient‐derived stem cells with CRISPR/Cas9‐mediated genome editing, this system allows direct assessment of mutation‐specific effects on *SERPING1* expression, intracellular processing, and C1INH secretion while maintaining endogenous genomic regulation. Through reciprocal editing experiments, we further demonstrated that these variants are sufficient to drive the observed molecular phenotypes.

One major achievement of this study is the efficient and reproducible generation of EPSCs from HAE patients together with their successful differentiation into HLCs. The resulting EPSC‐HLCs recapitulated key transcriptomic, proteomic, and functional characteristics of human hepatocytes, including ALB secretion and urea production. Importantly, these cells also reproduced key aspects of physiological C1INH biosynthesis and secretion. Secreted C1INH displayed a molecular weight comparable to plasma‐derived C1INH, although at lower absolute levels than PHHs. In contrast, HepG2 cells showed undetectable C1INH expression under our experimental conditions, highlighting the limitations of commonly used immortalized cell lines for studying hepatocyte‐specific aspects of HAE biology. Collectively, these findings establish the EPSC‐HLC system as a physiologically relevant platform for investigating liver‐specific mechanisms underlying HAE‐C1INH and provide a foundation for examining how *SERPING1* variants affect hepatocyte biology during differentiation and maturation.

Using this platform, we found that patient‐derived HLCs faithfully recapitulated the hallmark reduction of C1INH observed in HAE‐C1INH‐Type1. Compared with HCs, patient‐derived HLCs exhibited markedly reduced *SERPING1* transcript levels together with decreased secretion of C1INH protein. Notably, this phenotype emerged specifically during the mature hepatocyte stage and was not observed in undifferentiated EPSCs, definitive endoderm, or hepatic progenitor stages. These findings suggest that the pathogenic consequences of *SERPING1* variants are highly dependent on hepatocyte maturation state and further emphasize the importance of mature hepatocyte models for studying HAE pathophysiology.

To further investigate the molecular basis underlying reduced *SERPING1* expression in patient‐derived HLCs, we performed ASE analysis using bulk RNA sequencing data. We observed that mutant *SERPING1* alleles were consistently expressed at substantially lower levels than wild‐type (WT) alleles across all patient samples, including patient P1, who harbors a missense mutation that does not introduce a premature stop codon. This suppression was reproducible across multiple independent HLC differentiation experiments, suggesting that reduced mutant allele expression represents a robust and reproducible feature of these patient‐derived hepatocytes.

Interestingly, this mutant allele suppression was highly context dependent. When the identical P1 mutation was introduced into a healthy control background, the mutant allele was expressed at markedly higher levels. This striking reversal suggests that genetic background may substantially influence allele‐specific expression of *SERPING1* variants. Similar allele‐biased expression patterns have been reported in other inborn errors of immunity (IEIs) [32], supporting the concept that patient‐specific regulatory mechanisms may shape disease phenotypes beyond the pathogenic variant itself. Together, these findings suggest that differences in transcriptional regulation, RNA stability, or RNA processing may contribute to suppression of mutant *SERPING1* alleles in a patient‐specific manner.

Despite this shared pattern of mutant allele suppression, distinct *SERPING1* variant classes exhibited divergent intracellular phenotypes. In patients P1‐P3, including missense and frameshift variants, reduced *SERPING1* transcription and decreased C1INH secretion occurred without prominent intracellular accumulation of C1INH during HLC maturation. This observation differs from previous overexpression‐based studies in non‐hepatic systems, where certain *SERPING1* variants were proposed to exert dominant‐negative effects through intracellular aggregation and retention of C1INH protein [12, 13]. Our findings suggest that these previously reported retention phenotypes may not universally occur under endogenous expression conditions and highlight the importance of studying pathogenic variants within their native genomic and hepatocyte‐specific contexts.

In contrast, patient P4, who carries a large deletion spanning exons 1–4, exhibited an apparently distinct phenotype characterized by intracellular accumulation of C1INH together with impaired secretion and reduced hepatocyte maturation. This observation was unexpected, as gross deletions are generally presumed to result primarily in haploinsufficiency. Under our experimental conditions, no alternative C1INH isoforms were detected, and the observed C1INH migrated at a molecular weight similar to wild‐type protein, suggesting that the detected signal likely originated predominantly from the intact allele. Although the precise mechanism underlying this intracellular accumulation remains unclear, these findings further support the concept that distinct classes of *SERPING1* variants may produce heterogeneous molecular consequences at the hepatocyte level.

This mechanistic heterogeneity may also have important implications for understanding the clinical variability observed in HAE [33]. Although genotype–phenotype correlations in HAE remain incompletely defined, several studies have reported associations between mutation classes, residual C1INH levels, and disease severity [34]. In some cohorts, variants predicted to cause greater disruption of C1INH structure or expression, such as nonsense or frameshift mutations, have been associated with earlier onset or more severe clinical manifestations, whereas certain missense variants have been linked to milder phenotypes [34]. However, these correlations remain inconsistent across studies, and substantial variability is frequently observed even among individuals carrying identical mutations [3, 9]. The allele expression reversal observed in our P1 isogenic HLCs further supports the idea that additional genetic and regulatory factors may substantially influence disease expression beyond the pathogenic variant itself.

These observations further emphasize the complexity of linking individual *SERPING1* variants to clinical manifestations and highlight the need for mechanistic human models capable of dissecting hepatocyte‐intrinsic effects independently of systemic disease processes. While the current model does not capture the full systemic complexity of HAE, it provides a controlled framework for investigating the direct consequences of *SERPING1* variants without confounding downstream vascular, immune, and kallikrein‐kinin interactions. Importantly, this platform is not intended to replace current diagnostic approaches, which remain based primarily on biochemical assessment of C1INH levels and function [2]. Instead, its primary value lies in mechanistic investigation of variant‐specific effects, evaluation of variants of uncertain significance, and development of precision therapeutic strategies.

Such mechanistic insight may also inform future therapeutic development. Current HAE therapies primarily target the kallikrein‐bradykinin pathway and have significantly improved disease management [2, 8]. However, most emerging gene‐based approaches similarly focus on upstream pathway modulation rather than direct correction of *SERPING1* deficiency [8]. Our results demonstrate that mutation‐specific correction of *SERPING1* can restore both transcript expression and C1INH secretion in a physiologically relevant human hepatocyte context, providing proof‐of‐concept for therapeutic strategies that directly address the underlying genetic defect. Although substantial translational challenges remain, including delivery efficiency, long‐term safety, and scalability [35, 36], these findings support continued exploration of *SERPING1*‐targeted precision therapies.

Finally, several limitations of this study should be acknowledged. First, our analysis was limited to four patient‐derived lines representing selected *SERPING1* variant classes. Larger and more diverse cohorts will be necessary to more comprehensively define mutation‐specific mechanisms and evaluate genotype–phenotype relationships. Second, the current platform focuses primarily on hepatocyte‐intrinsic processes and does not model the downstream systemic consequences of C1INH deficiency, including endothelial responses, immune signaling, and kallikrein‐kinin pathway activation. Accordingly, this system should be viewed as a complementary mechanistic platform rather than a complete representation of systemic HAE pathophysiology.

In summary, we have established a patient‐derived EPSC‐HLC model that faithfully recapitulates the transcriptional deficits, secretory impairments, and intracellular protein accumulation caused by diverse *SERPING1* mutations in HAE. Unlike conventional systems, our platform retains the patient's complete genomic context, enabling precise, mutation‐specific mechanistic insights within a personalized framework. While demonstrated here for HAE, this approach is broadly applicable to other inherited liver disorders and holds significant promise for variant interpretation, therapeutic screening, and the future development of autologous cell‐based therapies using genetically corrected hepatocytes.

## Author Contributions

Xueyan Liu and Yuxin Wang contributed equally to this work and share first authorship. Xueyan Liu designed the study, performed the experiments, analyzed and interpreted the data, prepared the figures, and contributed to manuscript preparation. Yuxin Wang interpreted the data, prepared the graphical abstract, drafted the manuscript, and managed the submission process. Jane C.Y. Wong provided patient clinical data and drafted portions of the initial manuscript. Xiang Zhang conducted the protein structural and bioinformatics analyses, and contributed to the Western blot experiments. Cuixi Wu, Pengyu Chen, and Yunzhi Yang assisted with experiments, data collection, and technical support. Pentao Liu, Chak Sing Lau, and Matthew Cook contributed to scientific discussion and critical interpretation of the data. Philip Hei Li supervised the study, acquired funding, and contributed to study design, data interpretation, and manuscript preparation. All authors critically revised the manuscript and approved the final version for publication.

## Funding

This study was supported by the InnoHK initiative of the Innovation and Technology Commission of the Hong Kong Special Administrative Region Government.

## Conflicts of Interest

The authors declare no conflicts of interest.

## Supporting information


**Figure S1:** HAE Patient EPSC generation. (A) Schematic diagram of EPSC reprogramming. (B) Representative images of PBMC‐expanded erythroblasts from each patient. (C) Erythroblasts marker analysis by flow cytometry in PBMCs and PBMC‐expanded erythroblasts. (D) Representative images of reprogrammed EPSC colonies from each patient. (E) Trilineage marker expression in embryoid bodies derived from EPSCs, assessed by immunofluorescence staining.

**Figure S2:** Stage‐specific marker gene expression during EPSC to HLC differentiation. Pluripotent (A), definitive endoderm‐specific (B), hepatic progenitor (C), and mature hepatocyte (D) marker gene expression were assessed at Day 0, 5, 10, and 20 during HLC differentiation by qRT‐PCR analysis. HC, healthy control.

**Figure S3:** Representative significantly enriched KEGG pathways in EPSC‐derived HLCs compared to EPSCs, along with gene expression patterns across cell types. Gene sets and associated genes are shown for: (A) fat and cholesterol metabolism, (B) xenobiotic metabolism and detoxification, (C) bile secretion, (D) complement and coagulation cascades, and (E) drug metabolism. HC, healthy control; HC‐HLCs, healthy control‐derived HLCs; P‐HLCs, patient‐derived HLCs; PHHs, primary human hepatocytes.

**Figure S4:** Defective *SERPING1* expression and C1INH secretion in patient EPSC‐derived HLCs. (A) Time‐course analysis of *SERPING1* expression during HLC differentiation from patient and HC EPSCs. (B) Relative fold change of *SERPING1* on Day 20 of HLC differentiation compared to baseline EPSCs. (C) Detection of secreted C1INH by Western blot in the supernatants of patient and HC HLCs derived from different batches, using ALB as a reference.

**Table S1:** List and sequence of oligonucleotides and primers used.
**Table S2:** List of antibodies used in this study.

## Data Availability

The data that support the findings of this study are available from the corresponding author upon reasonable request.
