# Supplementary material for: Modeling Hereditary Angioedema With Personalized Expanded Potential Stem Cell‐Derived Hepatocytes: A CRISPR‐Validated Platform for Mutation‐Specific Mechanisms and Therapeutic Innovation
Source: Allergy. 2026 Jun 20;81(8):2858–73. doi: 10.1111/all.70419 (PMC13466179; doi:10.1111/all.70419)
Supplement: Supplementary file 3 [file ALL-81-2858-s004.pdf]

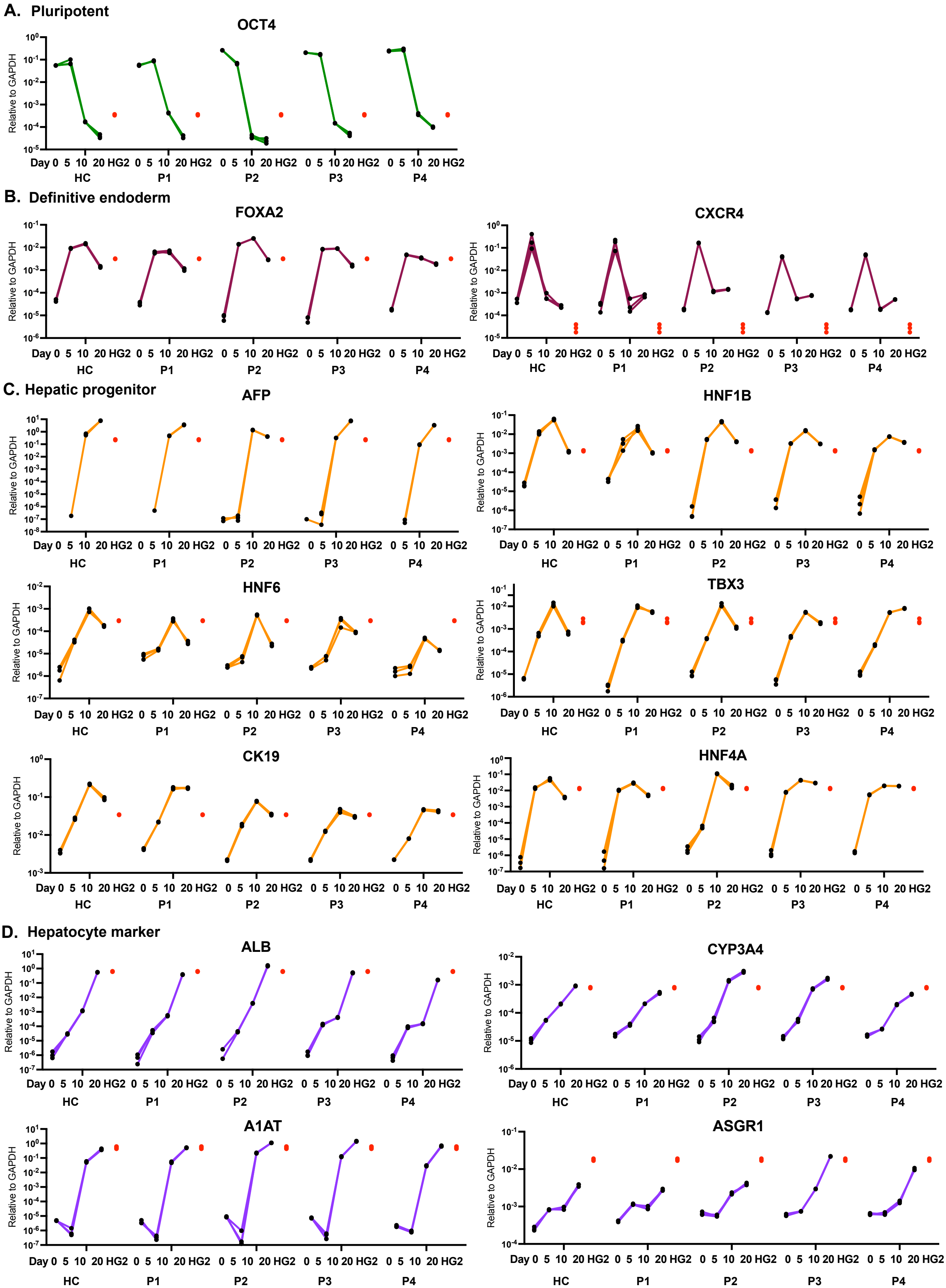

Supplementary Figure 2. Xueyan Liu et al.
