# Supplementary material for: Modeling Hereditary Angioedema With Personalized Expanded Potential Stem Cell‐Derived Hepatocytes: A CRISPR‐Validated Platform for Mutation‐Specific Mechanisms and Therapeutic Innovation
Source: Allergy. 2026 Jun 20;81(8):2858–73. doi: 10.1111/all.70419 (PMC13466179; doi:10.1111/all.70419)
Supplement: Supplementary file 5 [file ALL-81-2858-s001.docx]

**Supplementary Table 1: List and sequence of oligonucleotides and primers used**

| **Name** | **Description** | **Sequence** |
| --- | --- | --- |
| *GAPDH* F | qPCR primer F | CCTCAACGACCACTTTGTCAAG |
| *GAPDH* R | qPCR primer R | TCTTCCTCTTGTGCTCTTGCTG |
| *SERPING1* F1 | qPCR primer F | GCATCAAAGTGACGACCAGCCA |
| *SERPING1* R1 | qPCR primer R | GTCTCTGTCAGTTCCAGCACTG |
| *SERPING1* F2 | qPCR primer F | TCTTTGAAGTGCAGCAGCCC |
| *SERPING1* R2 | qPCR primer R | TCAGGCCCTGGGGTCATAT |
| *OCT4* F | qPCR primer F | CCTCACTTCACTGCACTGTA |
| *OCT4* R | qPCR primer R | CAGGTTTTCTTTCCCTAGCT |
| *FOXA2* F | qPCR primer F | GGAACACCACTACGCCTTCAAC |
| *FOXA2* R | qPCR primer R | AGTGCATCACCTGTTCGTAGGC |
| *CXCR4* F | qPCR primer F | CTCCTCTTTGTCATCACGCTTCC |
| *CXCR4* R | qPCR primer R | GGATGAGGACACTGCTGTAGAG |
| *AFP* F | qPCR primer F | GCAGAGGAGATGTGCTGGATTG |
| *AFP* R | qPCR primer R | CGTGGTCAGTTTGCAGCATTCTG |
| *HNF1B* F | qPCR primer F | CCCAGCAAATCTTGTACCAGGC |
| *HNF1B* R | qPCR primer R | ACCTCAGTGACCAAGTTGGAGC |
| *HNF6* F | qPCR primer F | GAGGATGTGGAAGTGGCTGCAG |
| *HNF6* R | qPCR primer R | CTGTGAAGACCAACCTGGGCTT |
| *TBX3* F | qPCR primer F | GGACACTGGAAATGGCCGAAGA |
| *TBX3* R | qPCR primer R | GCTGCTTGTTCACTGGAGGACT |
| *CK19* F | qPCR primer F | AGCTAGAGGTGAAGATCCGCGA |
| *CK19* R | qPCR primer R | GCAGGACAATCCTGGAGTTCTC |
| *HNF4A* F | qPCR primer F | GGTGTCCATACGCATCCTTGAC |
| *HNF4A* R | qPCR primer R | AGCCGCTTGATCTTCCCTGGAT |
| *ALB* F | qPCR primer F | GATGAGATGCCTGCTGACTTGC |
| *ALB* R | qPCR primer R | CACGACAGAGTAATCAGGATGCC |
| *CYP3A4* F | qPCR primer F | CCGAGTGGATTTCCTTCAGCTG |
| *CYP3A4* R | qPCR primer R | TGCTCGTGGTTTCATAGCCAGC |
| *A1AT* F | qPCR primer F | TCTGAAGAGCGTCCTGGGTCAA |
| *A1AT* R | qPCR primer R | GATGGTCAGCACAGCCTTATGC |
| *ASGR1* F | qPCR primer F | GAAGCAGTTCGTGTCTGACCTG |
| *ASGR1* R | qPCR primer R | AGCGAGAGAACCAGTAGCAGCT |
| sgRNA1 | P1 *SERPING1* mutation repair sgRNA | CAAGCAGGGTCTTACTGAGC |
| ssODN1 | P1 *SERPING1* mutation repair ssODN | GTGACTCTTATGGGAGTGTCCAACAAATGACCTGGAGAGAATTCAACCAGGGTCTTACCGAGCAGGACCTGGGTAAGGAGGCTGGCGATGCTGAATGGGGAAAAGGCCATGTTGGTCTCC |
| sgRNA2 | P2 *SERPING1*mutation repair sgRNA | TCAAAGACCCAGCAGGGTGC |
| ssODN2 | P2 *SERPING1* mutation repair ssODN | TGTGCTGCTGGTCCCAGAGCACGAAGAGGAAGGGCTGCTGCACTTCAAAGACCAGCAGGGTGCGAGCCACAGAGATGGCGGAGGCTGCAGCCGCCTCCACCCCAGTCTCTGTCAGTTCCA |
| sgRNA3 | gRNA for P1 *SERPING1* mutation installation on HC | CAAGCAGGGTCTTACCGAGC |
| ssODN3 | ssOND for P1 *SERPING1* mutation installation on HC | GTGACTCTTATGGGAGTGTCCAACAAATGACCTGGAGAGAATTCAACCAGGGTCTTACTGAGCAGGACCTGGGTAAGGAGGCTGGCGATGCTGAATGGGGAAAAGGCCATGTTGGTCTCC |
| sgRNA4 | gRNA for P2 *SERPING1* mutation installation on HC | CTGCACTTCAAAGACCAGCA |
| ssODN4 | ssOND for P2 *SERPING1* mutation installation in healthy controls (HCs) | GGAACTTGTGCTGCTGGTCCCAGAGCACGAAGAGGAAGGGCTGCTGCACTTCAAAGACCGAGCAGGGTGCGGGCCACAGAGATGGCGGAGGCTGCAGCCGCCTCCACCCCAGTCTCTGTCA |
| P1 gDNA aF | P1 *SERPING1* mutation amplify primer F | ACATCCACACCTTCTCTTCC |
| P1 gDNA aR | P1 *SERPING1* mutation amplify primer R | TTACAGCTTCTTCCCAGAGG |
| P1 gDNA sF | P1 *SERPING1* mutation sequence primer F | GACTGTGCCTCGTAGTAAGA |
| P1 gDNA sR | P1 *SERPING1* mutation sequence primer R | TTGTAAGTGTCTGGATTGGTG |
| P2 gDNA asF | P2 *SERPING1* mutation amplify and sequence primer F | TGTAATCTGGCAAACAAGGG |
| P2 gDNA asR | P2 *SERPING1* mutation amplify and sequence primer R | AGGGCTAATAGAAGCAGGTC |
| P3 gDNA asF | P3 *SERPING1* mutation amplify and sequence primer F | AGATGATCTGTGATCCCCTC |
| P3 gDNA asR | P3 *SERPING1* mutation amplify and sequence primer R | CTATTTTACTGTACCTGCCCG |
| P4 gDNA asF | P4 *SERPING1* mutation amplify and sequence primer F | CCTGGTCTCAAACTCCTGAT |
| P4 gDNA asR | P4 *SERPING1* mutation amplify and sequence primer R | GGCAGGAGAATCACTTGAATC |

**Supplementary Table 2. List of antibodies used in this study.**

| **Antibody** | **Source** | **Catalog** | **Purpose** |
| --- | --- | --- | --- |
| **Primary antibody** |  |  |  |
| Anti-human CD71 PE | Miltenyi Biotec | 130-115-071 | Erythroblast Flow |
| Anti-human CD235a FITC | Miltenyi Biotec | 130-117-800 | Erythroblast Flow |
| Mouse anti-OCT4 AF488 | Millipore | MAB4419A4 | EPSC IF |
| Mouse anti-SOX2 Cy3 | Millipore | MAB4423C3 | EPSC IF |
| Rabbit anti-Mouse/Human NANOG | Stemgent | 09-0020 | EPSC IF |
| Anti-beta III Tubulin AF594 | Abcam | ab201740 | Ectoderm IF |
| Anti-alpha smooth muscle Actin FITC | Abcam | ab223920 | Mesoderm IF |
| Mouse anti-Human SOX17 | R&D systems | MAB19241 | Endoderm IF |
| Rabbit anti-EpCAM AF594 | Abcam | ab286811 | HLC IF |
| Rabbit anti-ALB AF488 | Abcam | ab313348 | HLC IF & Flow |
| Rabbit anti-A1AT AF647 | Abcam | ab206735 | HLC IF & Flow |
| Mouse anti-Human Serpin G1/C1 | R&D Systems | MAB2488 | *SERPING1* WB |
| Rabbit anti-β-actin | Cell signaling | 4967S | β-actin WB |
| Goat anti-ALB-HRP | Thermo Fisher Scientific | A80-229P | ALB WB |
| **Secondary antibody** |  |  |  |
| Donkey anti-Rabbit IgG AF647 | Invitrogen | A-31573 | EPSC IF |
| Donkey anti-Mouse IgG AF647 | Invitrogen | A-31571 | Endoderm IF |
| Goat anti-Rabbit IgG HRP | Thermo Fisher Scientific | 31460 | β-actin WB |
| Goat anti-Mouse IgG HRP | Thermo Fisher Scientific | 31430 | *SERPING1* WB |
